# Impact of Early ARV Initiation on Relative Proportions of Effector and Regulatory CD8 T Cell in Mesenteric Lymph Nodes and Peripheral Blood During Acute SIV Infection of Rhesus Macaques

**DOI:** 10.1128/jvi.00255-22

**Published:** 2022-03-21

**Authors:** Alexis Yero, Omar Farnos, Julien Clain, Ouafa Zghidi-Abouzid, Henintsoa Rabezanahary, Gina Racine, Jérôme Estaquier, Mohammad-Ali Jenabian

**Affiliations:** a Department of Biological Sciences and CERMO-FC Research Centre, Université du Québec à Montréal (UQAM), Montreal, Quebec, Canada; b Centre Hospitalier Universitaire (CHU) de Québec Research Center, Faculty of Medicine, Université Laval, Quebec City, Quebec, Canada; Emory University

**Keywords:** gut mucosal immunity, CD8 T cells, CD8 regulatory T cells (CD8 Treg), early antiretroviral therapy, GALT, gut, human immunodeficiency virus (HIV), simian immunodeficiency virus (SIV)

## Abstract

CD8 T cells are key players in the clearance of human immunodeficiency virus (HIV)-infected cells, such that CD8 T-cell dysfunction contributes to viral persistence despite antiretroviral (ARV) therapy. Mesenteric lymph nodes (MLNs) are major sites of gut mucosal immunity. While different CD8 T cell subsets such as CD8 alpha-alpha (CD8αα), CD8 alpha-beta (CD8αβ), CD8 regulatory T cells (Treg), and mucosa-associated invariant T cells (MAIT) are present in the gut and exhibit distinct functions, their dynamics remain poorly understood due to the lack of accessibility to these tissues in humans. We thus assessed CD8 T cells in MLNs versus peripheral blood in simian immunodeficiency virus (SIV)-infected rhesus macaques (RMs) following early ARV therapy initiation. SIV infection was associated with an increase over time of both CD8αβ and CD8αα T cells in the blood and MLNs, whereas early ARV initiation significantly decreased the frequencies of CD8αα but not CD8αβ T cells in MLNs. A significant decrease in the expression of chemokine receptors CCR6 and CXCR3 by CD8 T cells, which are essential for T-cell trafficking to the inflammatory sites, was observed in chronically SIV-infected RMs. Surprisingly, while MAIT cells are increased in ARV-treated RMs, their frequencies in MLN are extremely low and were not impacted by ARV. The acute infection resulted in an early CD39^+^FoxP3^+^ CD8 Tregs increase in both compartments, which was normalized after early ARV. Frequencies of CD8 Treg cells were positively correlated with frequencies of CD4 Tregs and accordingly negatively correlated with the Th17/Treg ratio in the blood but not in MLNs. Overall, our results underscore the difference in CD8 T-cell subset dynamics in the blood and MLNs.

**IMPORTANCE** Changes in CD8 T-cell subsets during acute SIV/HIV infections and following early ARV initiation in gut lymphoid tissues are poorly understood. Using an acute SIV infection model in rhesus macaques, we assessed the impact of early ARV, initiated 4 days postinfection, on relative proportions of CD8 T-cell subsets in MLNs compared to blood. We found that acute SIV infection and early ARV initiation differentially affect the distribution of effector CD8 T cells, CD8 MAIT cells, and CD8 Tregs in MLNs compared to blood. Overall, early ARV initiation maintains the frequency of effector CD8 T cells while reducing immunosuppressive CD39^+^ CD8 Tregs. Our study provides deeper insight into the dynamics of the CD8 T-cell compartment in gut mucosal immune surveillance during acute SIV infection and following early ARV initiation.

## INTRODUCTION

CD8 T cells are essential in controlling simian immunodeficiency virus (SIV)/human immunodeficiency virus (HIV) infections. The expansion of HIV-specific CD8 T cells precedes the decrease of viremia during acute infection, suggesting that CD8 T cells are involved in the initial control of the infection; as such, HIV-specific CD8 T-cell expansion is a predictor of slow HIV disease progression (1–3). During SIV/HIV infections, persistent antigen exposure promotes a progressive dysfunction and exhaustion of CD8 T cells, associated with impaired proliferation, decreased cytokine production and cytolytic capacity, and cell death (2–7). Long-term antiretroviral (ARV) therapy partially restores CD8 T-cell functions, cytotoxicity, and proliferative capabilities (2–5), while a strong HIV-specific CD8 T-cell response is needed to suppress viral replication during ARV therapy (1, 8). Accordingly, CD8 T-cell depletion in SIV-infected rhesus macaques (RMs) under ARV results in increased viral loads due to loss of viral control (3, 9).

The CD8 receptor is expressed in 2 forms, either as the alpha-alpha (αα) homodimer or the alpha-beta (αβ) heterodimer. CD8αβ is a T-cell receptor (TCR) coreceptor which is essential for CD8 development and function, while the function of CD8αα is largely unknown. Differences in sensitivity to antigen, the efficiency of activation, and the stage of CD8 differentiation have been reported for each isoform (10–12). The αα homodimer is mainly expressed by terminal-differentiated CD8 T cells, a population with lower cytotoxicity capacity and poor antiviral response that is likely to be unable to control the viral replication. At the same time, the αβ heterodimer has been associated with a more functional phenotype (10–12).

Regulatory CD8 T cells (CD8 Tregs), which expressed FoxP3, the master regulator of Tregs, have immunosuppressive potential in cancer, autoimmune diseases, transplantation, and infectious diseases (13–17). Lim et al. showed that CD4^+^ and CD8^+^ T cells expressing FoxP3 in HIV-infected individuals are phenotypically distinct, while their frequencies increase with immune activation and remain elevated despite ARV (18). Previous reports suggested differential dynamics of CD8 Tregs during SIV/HIV infections according to the tissue localization. A rapid expansion of CD8 Tregs in colorectal mucosal tissues of SIV-infected RMs and the blood of HIV-infected individuals was reported (19), while another study did not find any increase in peripheral lymph nodes during SIV infection (20). CD8 Treg expansion correlated directly with SIV viremia and inversely with the magnitude of the antiviral response of SIV-specific interferon gamma-positive (IFN-γ^+^) T cells (19, 20). Notably, vaccine-induced CD8 Tregs were essential in controlling SIV infection in RMs due to their ability to suppress CD4^+^ T-cell activation and SIV replication (21, 22). Similarly, in HIV elite controllers, CD8 Tregs contribute to the natural control of HIV replication (23). Finally, increased frequencies of CD8 Tregs expressing the immunosuppressive ectonucleotidase CD39 correlated with HIV viremia, and immune activation markers have been reported (24). CD39, in orchestration with ectonucleotidase CD73, hydrolyzes inflammatory ATP into immunosuppressive adenosine (25–27). We and others reported that upregulation of CD39/adenosine pathways is associated with HIV disease progression, while CD39^+^ CD4 Treg levels in HIV-infected individuals, even under suppressive ARV, remain higher than uninfected individuals (28–31). However, CD8 Treg dynamics during acute SIV/HIV infections have been scarcely assessed, notably in mesenteric lymph nodes (MLNs).

Mucosa-associated invariant T (MAIT) cells are another unconventional mucosal CD8 T-cell subset, characterized by the expression of an invariant TCR Vα7.2 and CD161, which play a significant role in immune defense (32, 33). MAIT cells recognize conserved metabolite antigens from bacteria and fungi, making this population essential for controlling microbial infections (33). MAIT cells frequencies decrease during chronic HIV infection, whereas their dynamics in acute HIV/SIV infections and the impact of ARV on the MAIT cell frequencies remain controversial (33–37).

ARV initiation upon HIV exposure is highly recommended to facilitate CD4 T-cell recovery, improve clinical outcomes, and reduce the viral reservoir size (38, 39). Notably, early ARV initiation results in superior blood CD8 T-cell effector functions compared to ARV initiation in chronic infection (3, 5, 40, 41). However, to date, most of the research on CD8 T-cell function has been done in blood, while SIV/HIV replication occurs mainly in mucosal and lymphoid tissues such as the gut-associated lymphoid tissue (GALT) (39, 42). The use of migration markers such as CCR6 and CXCR3 allows us to distinguish those CD8 T cells with migratory potential to the gut and inflamed tissues (43, 44).

Importantly, early ARV initiation at 4 days after SIV infection was shown to suppress blood viral load (VL), whereas SIV persists within the MLNs of RMs (45). Interestingly, HIV-specific CD8 T cells in the lymph nodes and gut mucosa exhibit a weaker cytotoxic response in HIV-infected individuals than in blood (1, 46). However, the dynamics of effector and regulatory CD8 T cells in gut lymphoid tissues are understudied due to the limited access to these samples in HIV-infected individuals. We recently reported that very early ARV initiation during the acute SIV infection of RMs has a beneficial impact in restoring CD4 T-cells counts, decreasing immune activation, and preserving memory CCR6^+^ Th17 cells in both blood and MLNs of treated animals. However, early ARV initiation was unable to reduce the frequencies and homing of CD4 Tregs subsets and fibrosis markers within the MLNs. Again, these findings highlighted the differential dynamics of T-cell subsets in blood and MLNs during SIV/HIV infections (31). Here, in the same study cohort, we assessed dynamics of effector and regulatory CD8 T cell in MLNs versus in peripheral blood over time during SIV infection and following early ARV initiation.

(This work has been presented in part at the CROI 2020 Conference, Boston, MA, USA.)

## RESULTS

### Characteristics of study groups.

As described in Table 1, in our study, acute infection (early untreated) was considered up to 60 days of the infection (median, 33 days), while the group of chronically infected RMs included infected animals from 167 to 223 days (median, 191 days). Early ARV treatment was initiated 4 days after the infection (median of duration of ARV, 23.5 days). To evaluate the effect of ARV interruption, ARV was interrupted after 8 weeks of treatment in a group of early ARV-treated animals (median of time after ARV interruption, 15 days). As expected, a decrease in CD4 count per cubic millimeter compared to uninfected animals was observed in early untreated (median, 673/mm^3^ versus 1,056/mm^3^; Mann-Whitney *P* = 0.04) and chronically infected animals (median, 563 versus 1,056/mm^3^; Mann-Whitney *P* = 0.02), while early ARV treatment restored CD4 count, and ARV interruption did not affect CD4 count. Early untreated SIV infection increased plasma VL (median, 5.7 × 10^6^ copies/mL), while the early treatment at 4 days postinfection significantly decreased the VL to undetectable levels in 8 of the 9 early ARV-treated RMs (median of duration of ARV, 23.5 days). Of note, only one early-treated animal, which was sacrificed at day 11 postinfection and treated for 7 days, had a detectable VL (5.8 × 10^2^ copies/mL). A viral rebound in all early ARV-interrupted animals (median, 3.1 × 10^5^ copies/mL) occurred following ARV interruption.

**TABLE 1:** Virological and immunological characteristics of animals in each study group^*b*^

Infection or treatment status	Monkey code	Duration of infection (days)	Duration of ARV (days)	Time after ARV interruption (days)	Plasma viral load (copies/mL)	CD4 T-cell count/mm^3^ in blood	Specimen type^*c*^	
							Blood	MLNs
Uninfected	PB030	NA^*a*^	NA	NA	NA	1,084	Yes	No
	PB033	NA	NA	NA	NA	851	Yes	No
	PB036	NA	NA	NA	NA	1,027	Yes	No
	PB046	NA	NA	NA	NA	590	Yes	No
	PB049	NA	NA	NA	NA	457	Yes	No
	PB051	NA	NA	NA	NA	825	Yes	No
	PB061	NA	NA	NA	NA	1,715	Yes	No
	PB057	NA	NA	NA	NA	2,338	Yes	No
	9052732	NA	NA	NA	NA	1,325	Yes	Yes
	9071222	NA	NA	NA	NA	2,492	Yes	Yes
Early untreated	PB006	11	NA	NA	2.80E+07	1,044	Yes	No
	PB041	11	NA	NA	1.90E+06	555	Yes	No
	PB005	14	NA	NA	5.80E+06	473	Yes	No
	PB051	14	NA	NA	4.70E+07	917	Yes	No
	PB015	29	NA	NA	1.10E+06	1,300	Yes	Yes
	PB033	29	NA	NA	6.50E+06	673	Yes	Yes
	9051222	33	NA	NA	1.60E+06	636	Yes	Yes
	PB044	33	NA	NA	7.10E+05	738	Yes	No
	PB023	36	NA	NA	5.70E+06	415	Yes	No
	PB028	36	NA	NA	2.30E+06	553	Yes	No
	PB055	36	NA	NA	3.20E+07	1,261	Yes	Yes
	PB030	46	NA	NA	2.10E+07	61	Yes	Yes
	9082012	60	NA	NA	1.70E+04	805	Yes	Yes
Early ARV treated	R110806	11	7	NA	5.80E+02	1,393	Yes	Yes
	11-1466R	14	10	NA	0.00E+00	1,550	Yes	Yes
	R110804	14	10	NA	0.00E+00	1,912	Yes	No
	R110562	27	23	NA	0.00E+00	1,314	Yes	Yes
	11-1430R	28	24	NA	0.00E+00	3,496	Yes	No
	R110360	35	31	NA	0.00E+00	734	Yes	Yes
	R110482	35	31	NA	0.00E+00	2,945	Yes	No
	13-1660R	36	32	NA	0.00E+00	861	No	Yes
	12-1836R	55	51	NA	0.00E+00	922	Yes	Yes
Early ARV interrupted	R110482	72	56	12	1.10E+05	2,186	Yes	Yes
	12-1888R	72	56	12	7.66E+05	1,450	Yes	Yes
	R110804	75	56	15	3.71E+03	899	Yes	Yes
	12-1134R	75	56	15	3.49E+07	1,010	Yes	Yes
	11-1430R	78	56	18	3.08E+05	1,274	Yes	Yes
Chronic	PB023	167	NA	NA	1.39E+08	254	Yes	Yes
	PB013	188	NA	NA	3.57E+06	764	Yes	Yes
	PB028	194	NA	NA	1.61E+08	370	Yes	No
	PB044	223	NA	NA	5.71E+07	756	Yes	Yes

a NA, nonapplicable.

b Blood specimens from three untreated animals were assessed longitudinally in uninfected and early untreated groups (PB030, PB033, PB051), three other untreated animals were assessed longitudinally in both early untreated (acute) and chronic phases (PB023, PB028, PB044), and three early ARV-treated animals were assessed longitudinally in both early ARV-treated and early ARV-interrupted groups (R110482, R110804, 11-1430R).

c Yes represent that a sample was taken, while No represents that no sample was taken.

### Impact of early ARV on CD8αα and CD8αβ T-cell subsets.

**FIG 1:**
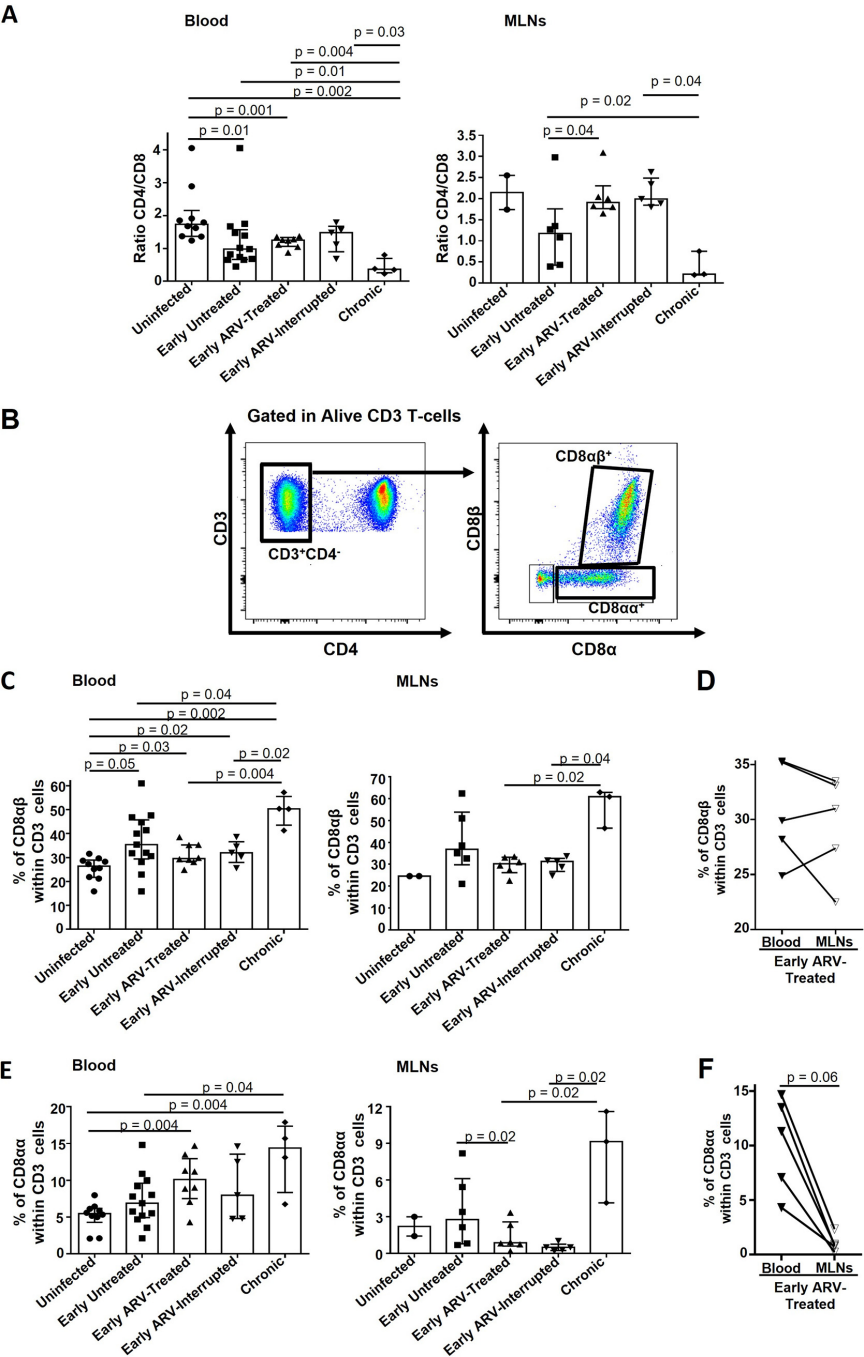
Impact of early ARV initiation on CD8 subsets during SIV infection. (A) CD4/CD8 ratio in both whole blood and MLNs. (B) Gating strategy used in flow cytometry to define CD8αβ T cells and CD8αα T cells in both whole blood and MLNs. (C) Percentages of CD8αβ T cells in both whole blood and MLNs. (D) Dynamics of CD8αβ T cells in matched blood versus MLNs of early ARV-treated animals. (E) Percentages of CD8αα T cells in both whole blood and MLNs. (F) Dynamics of CD8αα T cells in matched blood versus MLNs of early ARV-treated animals. Statistical significance is indicated in the figures. Differences among five study groups was determined by nonparametric Mann-Whitney rank test for unpaired variables, while the Wilcoxon rank tests were used for paired variables in the early ARV-treated group. Sample size in cross-sectional analysis as follows: uninfected, *n* = 10 in blood and *n* = 2 in MLNs; early untreated, *n* = 13 in blood and *n* = 6 in MLNs; early ARV treated, *n* = 8 in blood and *n* = 6 in MLNs; early ARV interrupted, *n* = 5 in blood and *n* = 5 in MLNs; and chronic, *n* = 4 in blood and *n* = 3 in MLNs. Sample size in paired analysis, *n* = 5.

The CD4/CD8 ratio is widely used as a clinical marker of disease progression during HIV infection since it is associated with clinical outcome, immune dysfunction, and viral reservoir size in the ARV-treated individuals (41, 47). As expected, we observed a decrease in the CD4/CD8 ratio during both acute (early untreated) and chronic stages of the disease in both blood and MLNs, while early ARV restored the CD4/CD8 ratio within the MLNs. ARV interruption did not affect the CD4/CD8 ratio in MLNs or blood, which can be consistent with the early sacrifice of ARV-interrupted animals (Fig. 1A).

Given that CD8 T cells expressing αα or αβ heterodimer may play different roles (10–12), we analyzed their dynamics (Fig. 1B). Untreated SIV infection increased, over time, the frequencies of CD8αβ in blood and MLNs, while no differences were observed in their percentages in the ARV-treated group (Fig. 1C). A higher percentage of CD8αα T cells was observed in chronically infected animals in both the blood and MLNs, whereas early ARV initiation only decreased their frequencies in the MLNs (Fig. 1E). The percentages of CD8αα and CD8αβ T cells after early ARV interruption were similar to the percentages observed in ARV-treated RMs (Fig. 1C and E). Only a correlation has been observed between the frequencies of CD8αα in MLNs with plasma VL (Table 2). To gain further insight into the distribution of CD8αα and CD8αβ in both compartments, we compared the available matched blood and MLN samples of early ARV-treated animals. Although there were no differences in CD8αβ frequencies between the blood and the MLNs (Fig. 1D), CD8αα frequencies were consistently higher in the blood than MLNs (Fig. 1F). Altogether, these results demonstrated that early ARV initiation differentially affects the frequencies of CD8αα and CD8αβ in blood and MLNs.

**TABLE 2:** Spearman correlations between the frequency of CD8 T-cell subsets and plasma VL and CD4 T-cell count^*a*^

CD8 T-cell subset	Spearman correlation with plasma VL copies/mL (all samples) for:				Spearman correlations with CD4 count/mm^3^ (all samples) for:			
	Blood		MLNs		Blood		MLNs	
	*r*	*P* value	*r*	*P* value	*r*	*P* value	*r*	*P* value
CD8αα	−0.1851	0.3274	**0.4297**	**0.05**	−0.0695	0.66	−0.3779	0.08
CD8αβ	0.313	0.09	0.4167	0.06	−0.3229	0.04	−0.2451	0.27
Memory CCR6^+^ CD8 T cells	−**0.4538**	**0.01**	−0.2986	0.2	0.1601	0.32	0.1175	0.6
Memory CXCR3^+^ CD8 T cells	−0.1612	0.39	−0.2303	0.32	−0.2552	0.11	−0.1304	0.56
MAIT CD8 T cells	−0.2921	0.11	−0.0076	0.9745	**−0.3406**	**0.03**	−0.0899	0.69
CD8^+^ FoxP3^+^	0.2648	0.15	0.1955	0.4	**−0.3055**	**0.05**	−0.1869	0.4
CD8^+^ FoxP3^+^ CD39^+^	**0.5309**	**0.002**	**0.5485**	**0.01**	**−0.5099**	**0.0008**	−0.3868	0.07

a Significant differences (*P* < 0.05) are highlighted in bold.

### Changes in frequencies of memory CCR6^+^ and CXCR3^+^ CD8 T cells in SIV-infected RMs.

**FIG 2:**
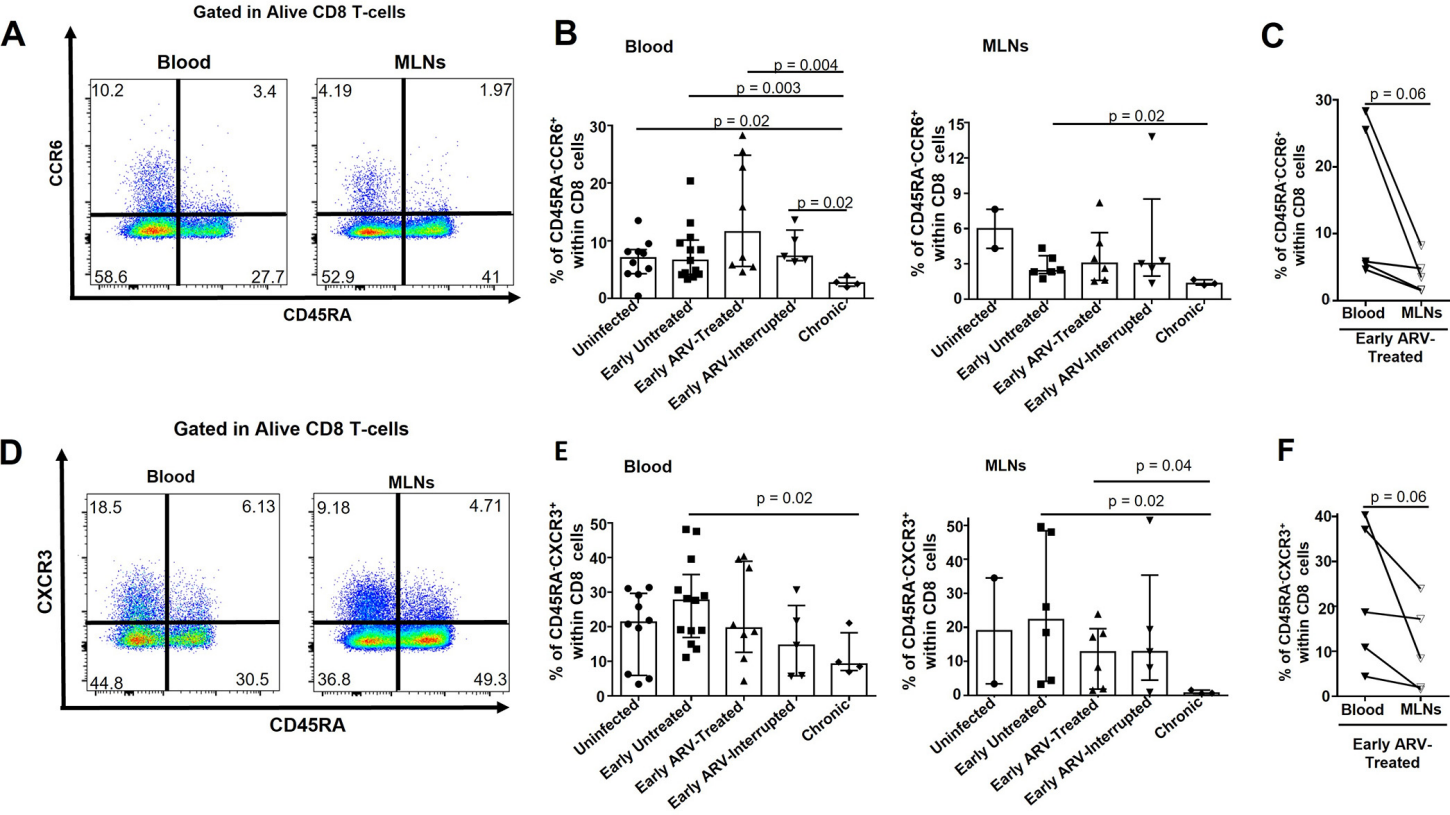
Effect of early ARV initiation on the expression of migration markers CCR6 and CXCR3 by memory CD8 T cells. (A) Gating strategy used in flow cytometry to define CCR6^+^ memory CD8 T cells in both whole blood and MLNs. (B) Percentages of CCR6^+^ memory CD8 T cells in both whole blood and MLNs. (C) Dynamics of CCR6^+^ memory CD8 T cells in matched blood versus MLNs of early ARV-treated animals. (D) Gating strategy used in flow cytometry to define CXCR3^+^ memory CD8 T cells in both whole blood and MLNs. (E) Percentages of CXCR3^+^ memory CD8 T cells in both whole blood and MLNs. (F) Dynamics of CXCR3^+^ memory CD8 T cells in matched blood versus MLNs of early ARV-treated animals. Statistical significance is indicated in the figures. Differences among five study groups were determined by nonparametric Mann-Whitney rank test for unpaired variables, while the Wilcoxon rank tests were used for paired variables in the early ARV-treated group. Sample size in cross-sectional analysis as follows: uninfected, *n* = 10 in blood and *n* = 2 in MLNs; early untreated, *n* = 13 in blood and *n* = 6 in MLNs; early ARV treated, *n* = 8 in blood and *n* = 6 in MLNs; early ARV interrupted, *n* = 5 in blood and *n* = 5 in MLNs; and chronic, *n* = 4 in blood and *n* = 3 in MLNs. Sample size in paired analysis, *n* = 5.

We then assessed the expression of chemokine receptors CCR6 and CXCR3 involved in the migration of T cells toward the gut and inflamed tissues (43, 44). Interestingly, our results indicated higher percentages of memory CXCR3^+^ CD45RA^−^ than CCR6^+^ CD45RA^−^ CD8 T cells in both compartments (Fig. 2). While the percentages of both memory CD8 T-cell subsets in acutely infected RMs (early untreated) are similar to those of uninfected animals (Fig. 2B and E), these percentages decreased considerably in chronically SIV-infected RMs and were particularly lower in MLN than the blood. Importantly, we noticed that the frequencies of CXCR3^+^ CD45RA^−^ CD8 T cells were highly divergent between animals, ranging from less than 5% to 50% in MLNs and the blood (Fig. 2E) in contrast to that observed for the CCR6^+^ CD45RA^−^ CD8 T-cell subset (Fig. 2B). Furthermore, the percentages of both populations were similar between animals in whom ARV was interrupted and those who remained on ARV. Only in blood, we found a negative correlation between memory CCR6^+^ CD45RA^−^ CD8 T cells and plasma VL (Table 2). In matched blood and MLN specimens of early ARV-treated animals, we observed consistently lower frequencies of both CCR6^+^ CD45RA^−^ and CXCR3^+^ CD45RA^−^ memory CD8 T cells in MLNs than blood (Fig. 2C and F). Our results indicate that CD8 T cells might decrease their migration to the gut and inflamed sites over time during SIV infection, while early ARV did not affect this ability.

### Early ARV initiation increases the frequencies of MAIT cells in the blood.

**FIG 3:**
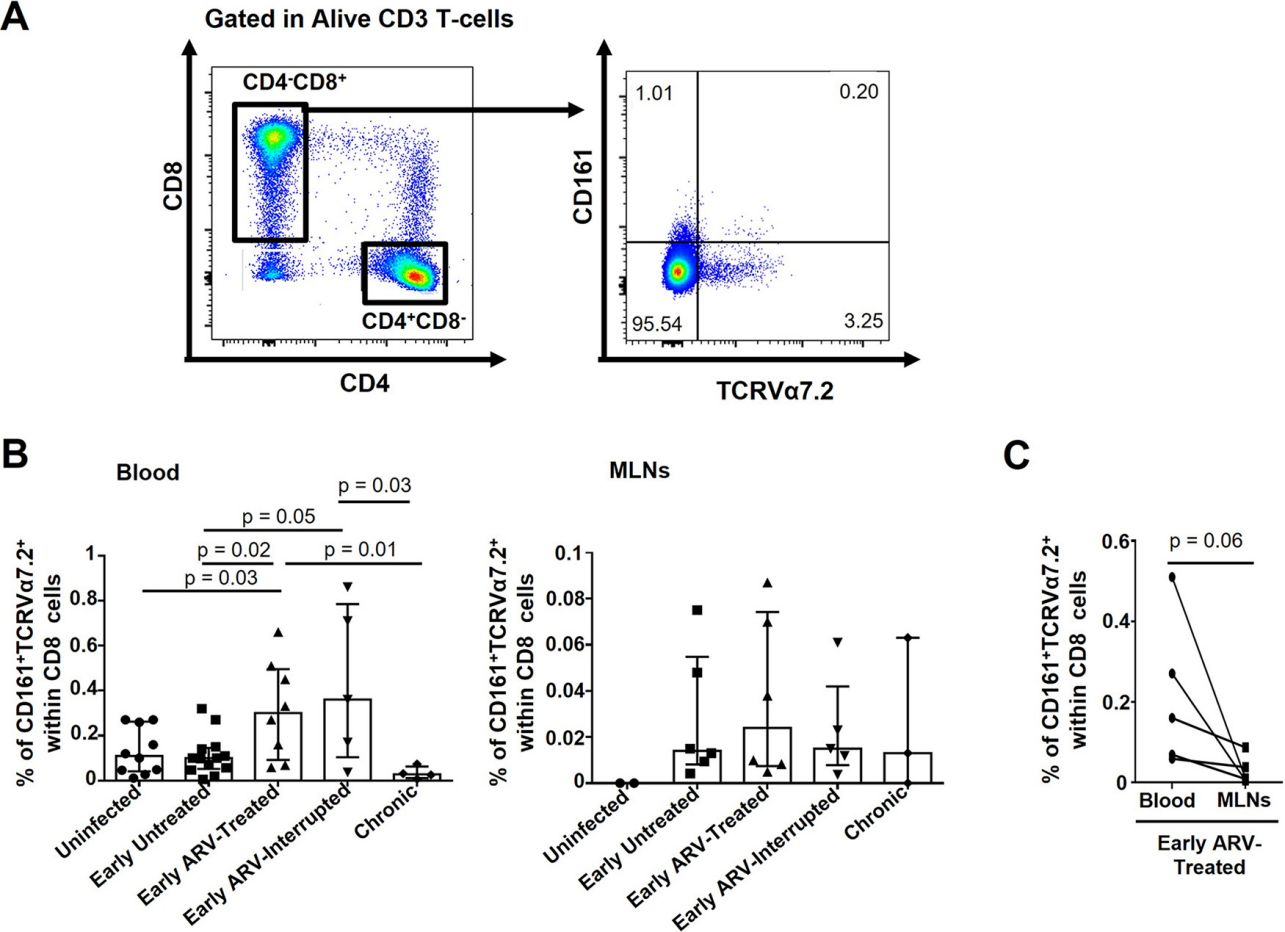
Effect of early ARV initiation on CD8 MAIT cells. (A) Gating strategy used in flow cytometry to define CD8 MAIT cells in both whole blood and MLNs. (B) Percentages of CD161^+^TCRVa7.2^+^ CD8 MAIT cells in both whole blood and MLNs. (C) Dynamics of CD161^+^ TCRVα7.2^+^ CD8 MAIT cells in matched blood versus MLNs of early ARV-treated animals. Statistical significance is indicated in the figures. Differences among five study groups was determined by nonparametric Mann-Whitney rank test for unpaired variables, while the Wilcoxon rank tests were used for paired variables in the early ARV-treated group. Sample size in cross-sectional analysis as follows: uninfected, *n* = 10 in blood and *n* = 2 in MLNs; early untreated, *n* = 13 in blood and *n* = 6 in MLNs; early ARV treated, *n* = 8 in blood and *n* = 6 in MLNs; early ARV interrupted, *n* = 5 in blood and *n* = 5 in MLNs; and chronic, *n* = 4 in blood and *n* = 3 in MLNs. Sample size in paired analysis, *n* = 5.

MAIT CD8 T cells are crucial to fight bacterial and fungal infections (32). Here, the frequencies of MAIT CD8^+^ CD161^+^ TRCVα7.2^+^ T cells in both the blood and MLNs were extremely low (less than 1% of the CD8 T cells) in uninfected RMs (Fig. 3A and B). We noticed a distinct dynamic between blood and MLNs. While the percentages of MAIT CD8 T cells remained stable in the blood during the acute infection (early untreated), they tend to increase in the MLNs during this stage (Fig. 3B). In addition, the percentages of this population in the blood increased in early ARV-treated and early ARV-interrupted RMs compared to uninfected animals, whereas they remained stable in MLNs (Fig. 3B). Only in blood, a negative correlation was observed between the frequencies of MAIT cells and CD4 T-cell count (Table 2). We observed lower frequencies of MAIT CD8 T cells in MLNs than blood in paired specimens in the early ARV-treated group (Fig. 3C). Taken together, our results demonstrated that frequencies of MAIT cells remained stable during SIV infection compared to uninfected animals in both compartments, while they increased in the blood of early ARV-treated SIV-infected RMs.

### Impact of early ARV on CD8 Tregs in blood and MLNs.

**FIG 4:**
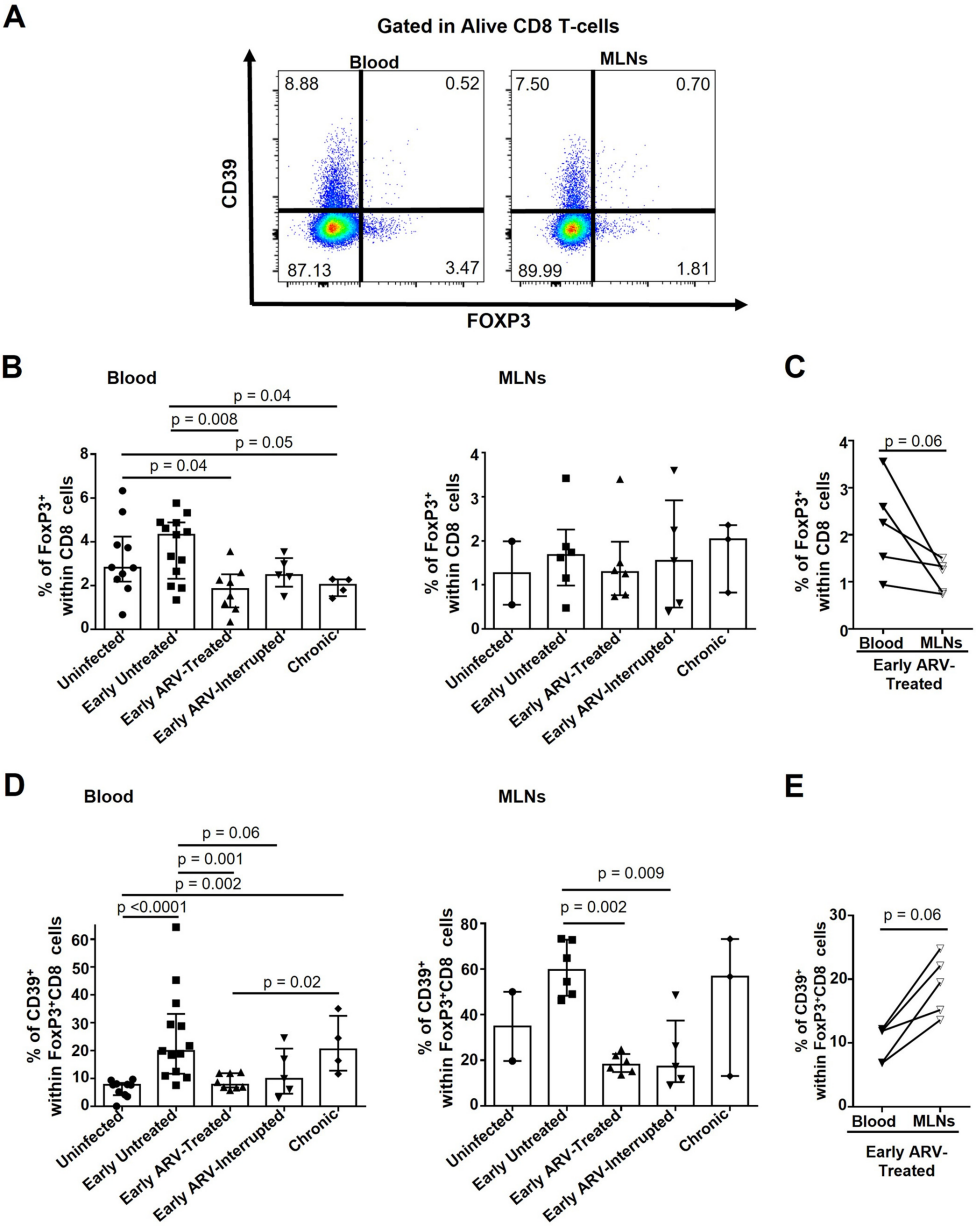
Effect of early ARV on total CD8 Tregs and CD39^+^ CD8 Tregs. (A) Gating strategy used in flow cytometry to define CD8 Tregs (FoxP3^+^ CD8 T cells) and CD39^+^ CD8 Tregs (CD8^+^ FoxP3^+^ CD39^+^) in both whole blood and MLNs. (B) Percentages of total CD8 Tregs in both whole blood and MLNs. (C) Dynamics of total CD8 Tregs in matched blood versus MLNs of early ARV-treated animals. (D) Percentages of CD39^+^ CD8 Tregs in both whole blood and MLNs. (E) Dynamics of CD39^+^ CD8 Tregs in matched blood versus MLNs of early ARV-treated animals. Statistical significance is indicated in the figures. Differences among five study groups was determined by nonparametric Mann-Whitney rank test for unpaired variables, while the Wilcoxon rank tests were used for paired variables in the early ARV-treated group. Sample size in cross-sectional analysis as follows: uninfected, *n* = 10 in blood and *n* = 2 in MLNs; early untreated, *n* = 13 in blood and *n* = 6 in MLNs; early ARV treated, *n* = 8 in blood and *n* = 6 in MLNs; early ARV interrupted, *n* = 5 in blood and *n* = 5 in MLNs; and chronic, *n* = 4 in blood and *n* = 3 in MLNs. Sample size in paired analysis, *n* = 5.

Initial reports have suggested that SIV/HIV infections are associated with increased CD8 Treg frequencies in the blood and colorectal mucosal tissue, contributing to disease progression (18–20). Furthermore, increased frequencies of immunosuppressive CD39^+^ CD8 Tregs are a marker of poor antiviral response (19, 24). First, we observed an increasing trend in total CD8 Treg frequencies in the blood of early untreated SIV-infected RMs (Fig. 4A and B). Chronic infection was associated with a significant decrease in total circulating blood CD8 Tregs compared with acutely infected (early untreated) and uninfected RMs (Fig. 4A and B). Their frequencies remained stable in MLNs following SIV infection (Fig. 4B). Of interest, early ARV decreased the frequencies of blood CD8 Tregs compared with early untreated SIV-infected RMs, while no effect was observed in the MLNs (Fig. 4B). In matched blood and MLN specimens of early ARV-treated animals, we observed consistently lower frequencies of total CD8 Tregs in MLNs than in the blood (Fig. 4C). Furthermore, our results indicated that SIV infection is associated with an increase in a subset of CD8 Tregs expressing CD39^+^ both in the blood and MLNs, while early ARV decreased their frequencies in both compartments (Fig. 4D). Only in blood, we observed negative correlations between CD8 Tregs and CD39^+^ CD8 Tregs with CD4 counts, while only for CD39^+^ CD8 Tregs, we detected a positive correlation with plasma VL in both blood and MLNs (Table 2). In matched blood and MLN specimens of early ARV-treated animals, we observed higher frequencies of CD39^+^ CD8 Tregs in MLNs than in the blood (Fig. 4E). Collectively, our results showed differential dynamics of CD8 Tregs in blood compared to MLNs, while early treatment normalized the frequencies of CD39^+^ CD8 Tregs in both compartments.

### CD8 Tregs are differently associated with CD4 Tregs and memory CCR6^+^ Th17/CD4 Treg ratio in blood compared to the MLNs.

**FIG 5:**
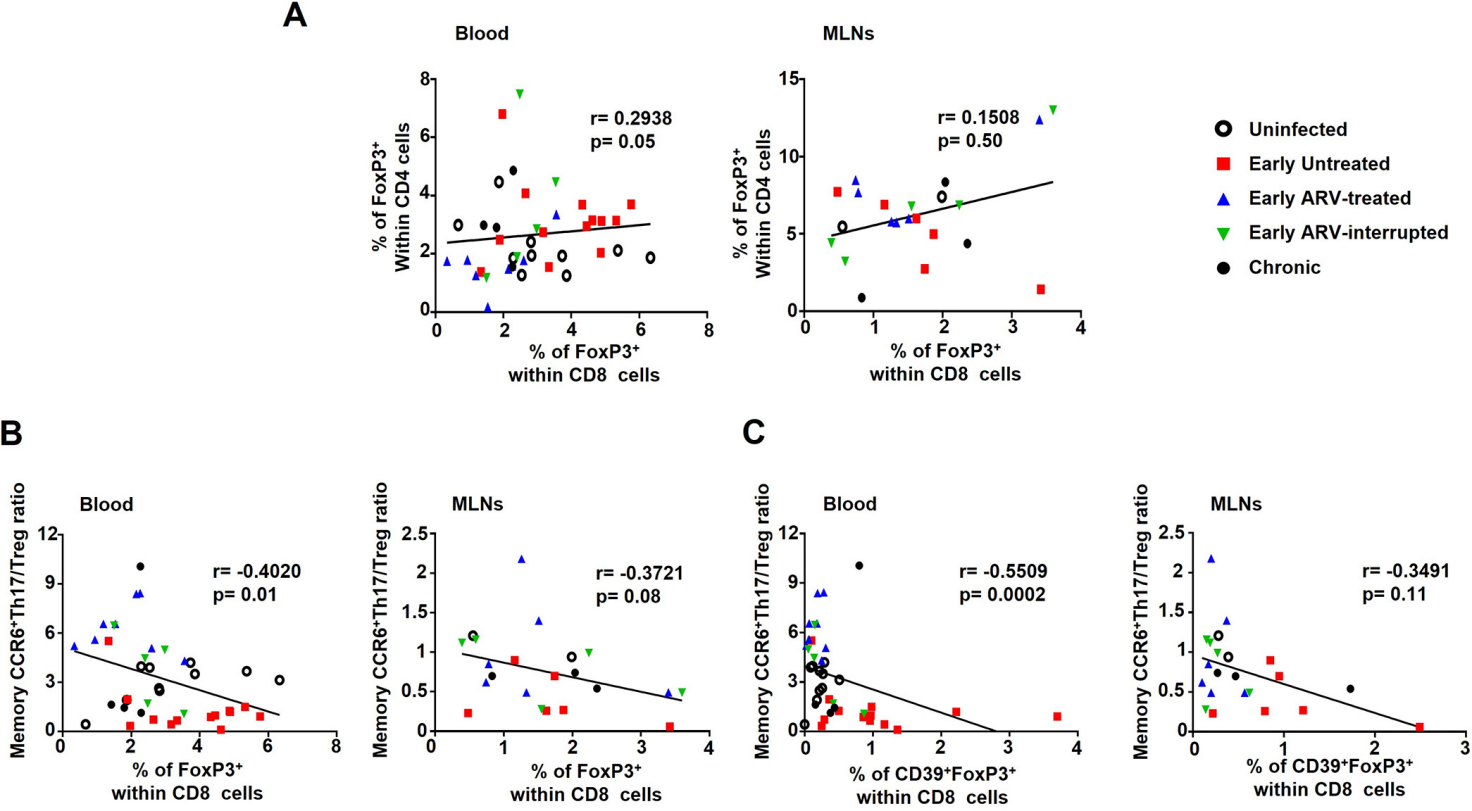
Correlation between CD8 Tregs and CD4 Tregs (A) and memory CCR6^+^ Th17/Treg ratio (B) in both blood and MLNs. (C) Correlation between CD39^+^ CD8 Tregs and memory CCR6^+^ Th17/Treg ratio in both blood and MLNs. Correlations were done with the following sample size: uninfected, *n* = 10 in blood and *n* = 2 in MLNs; early untreated, *n* = 13 in blood and *n* = 6 in MLNs; early ARV treated, *n* = 8 in blood and *n* = 6 in MLNs; early ARV interrupted, *n* = 5 in blood and *n* = 5 in MLNs; and chronic, *n* = 4 in blood and *n* = 3 in MLNs. *P* values were obtained following the Spearman correlation coefficient test.

The balance between Th17 cells and CD4 Tregs is commonly associated with better gut mucosal immunity and GALT functions. Higher CD8 Treg frequencies have also been associated with a decrease in Th17/Treg ratio in some inflammatory diseases (48, 49), but no reports of this relationship have been published in the context of SIV/HIV infections. We thus assessed the correlation between CD8 Treg frequencies and CD4 Tregs and memory CCR6^+^ Th17/Treg ratio on the same study cohort. Of note, all these data are presented in our previous publication (31). We observed a positive correlation between FoxP3^+^ CD8 Treg and FoxP3^+^ CD4 Treg frequencies in the blood only (Fig. 5A), while a negative association was detected between the memory CCR6^+^ Th17/Treg ratio and FoxP3^+^ CD8 Treg (Fig. 5B) and CD39^+^ FoxP3^+^ CD8 Treg (Fig. 5C) frequencies again in the blood only. Altogether, our results showed differential dynamics of total CD8 Tregs and CD39^+^ CD8 Tregs in blood compared to MLNs.

## DISCUSSION

The GALT is one of the main sites of SIV/HIV replication and immune dysfunction (39, 50). Consequently, an efficient and strong CD8 T-cell response in the GALT is necessary to control viral replication properly. However, our knowledge about CD8 T-cell dynamics during HIV infection in those deep tissues is limited due to the lack of accessibility in humans. Here, we have shown the impact of very early ARV initiation at 4 days post-SIV infection on the dynamics of different subsets of effector and regulatory CD8 T cells in the MNLs compared with the peripheral blood. Our results showed that early ARV initiation restores the CD4/CD8 ratio and maintains the frequencies of effector CD8 T cells within the MLNs while limiting the increase of immunosuppressive CD39^+^ CD8 Tregs within this tissue.

As expected, we observed a progressive decrease in the CD4/CD8 ratio in both blood and MLNs of SIV-infected animals during the early untreated and chronic infection stages. Very early ARV initiation played a beneficial role by restoring the CD4/CD8 ratio within the MLNs, but this restoration was less pronounced in the blood. Our team has previously shown that in acute SIV infection, levels of immune activation correlate with increased total CD8 frequencies (51, 52). To further assess CD8 subsets, here, we evaluated the dynamics of CD8αα and CD8αβ in the blood and the MLNs since these CD8 forms are rarely examined separately in SIV/HIV infection studies despite their distinct functions. CD8-expressing αβ subunits are efficient in eliminating pathological or damaged epithelial cells and fighting infection, while those expressing the αα exhibit higher tolerance to antigens in the gut (53). Here, we observed an increase in frequencies of CD8αα and CD8αβ T cells over time during SIV infection in the blood and the MLNs. The increase of CD8αα was anticipated, and it is in line with existing literature (11). However, our results differ from a previous report proposing a model system where viral protein nef downregulates the CD8β chain (54). Importantly, CD8 T cells expressing the αβ coreceptor are expected to suppress viral replication more effectively than those expressing the CD8αα form (10–12). The αα homodimer is mainly expressed by terminally differentiated CD8 T cells, displaying lower cytotoxic capacity and inadequate antiviral response (11). Furthermore, CD8αα functions as a TCR corepressor that negatively regulates cell activation (12). Thus, the observed decrease in the frequencies of CD8αα within the MLNs after early ARV initiation could improve CD8 T-cell viral control in this compartment.

We showed that during early untreated SIV infection, CD8 T cells retained the expression of both CCR6 and CXCR3, suggesting they maintained their migratory potential into MLNs. In line with our findings, no differences in CCR6^+^ CD8 T-cell frequencies between SIV/HIV-infected and uninfected subjects have been reported (55, 56). Similar to our data, increased frequencies of CXCR3^+^ CD8 T cells in both the blood and peripheral LNs have been reported during acute infection, which then decrease with disease progression (57, 58). In addition, Wacleche et al. proposed that CD8 T-cell recruitment to the GALT was mainly dependent upon integrin β7 and chemokine receptor CXCR3, while a limited CD4 and CD8 T-cell colocalization mediated by CCR6-dependent recruitment could contribute to poor viral control of the infection (59). Thus, in our context, the limited migration of CD8 T cells toward MLNs during acute SIV infection could negatively impact antiviral response within the GALT. Our observations of consistently lower frequencies of memory CCR6^+^ and CXCR3^+^ memory CD8 T cells in MLNs than the blood strongly support this hypothesis. This lower frequency may also reflect the observation that CD8 T cells within MLNs of SIV-infected RMS are prone to die, in relationship with high expression of transforming growth factor β1 (TGF-β1) (7, 60), and we reported an increase in TGF-β1 that that remained higher despite early ARV in this compartment (31).

MAIT cells remained stable in both blood and MLNs during early untreated and chronic SIV infection of RMs. To the best of our knowledge, this is the first time that the effect of very early ARV initiation on MAIT cell frequencies was evaluated. In line with previous reports on preservation and expansion of MAIT cells during acute HIV infection (36, 37), we observed increased MAIT cells after early ARV initiation only in the blood of treated animals, while their frequencies remained stable within the MLNs. Increased MAIT cell frequencies following early ARV interruption in blood could be due to their expansion during early untreated infection, which was swiftly inhibited by early ARV, while, following early ARV interruption and subsequent increase in the viral load, these cells could continue to expand.

Similar to our previous observations on CD4 Treg dynamics in the same study cohort (31), we found a distinct dynamic of CD8 Treg cells in the blood and MLNs. Thus, we detected a positive correlation between CD4^+^ and CD8^+^ Treg frequencies in the blood only. These findings are particularly important since CD4^+^ and CD8^+^ Tregs could use distinct and complementary immunosuppressive mechanisms. CD8 Tregs can be activated via MHC-I, which is virtually expressed by all cell types, while CD4 Tregs can be activated only by MHC-II-expressing cells. The cytokines produced by CD8 Tregs could also contribute to the stability and expansion of CD4 Tregs. TGF-β1 produced by CD8 Tregs promotes CD4 Treg expansion (61), while IL-10 is needed for FoxP3 expression (62). The CD8 Treg persistence and local expansion within the MLNs could also contribute to higher TGF-β1 levels and further tissue fibrosis within this compartment, as we previously showed in the same cohort (31). A previous study has reported a decrease in the frequency of CD8 Treg in MLNs of ARV-treated controller RMs (63). Our results indicated the absence of major difference in total CD8 Treg cells in MLNs, similar to a previous study reporting no changes in these cells in peripheral lymph nodes (20). However, CD8 Tregs expressing CD39 are clearly increased both in blood and MLNs of SIV-infected RMs and can be decreased by early ARV. The expression of ectonucleotidase CD39 by CD8 Tregs is crucial for viral suppression in SIV-infected RMs (19), and their levels are positively correlated with both disease progression and chronic immune activation (24). Here, we have shown that SIV infection and plasma VL were associated with increased immunosuppressive CD39^+^ CD8 Tregs, which were reduced by early ARV in both the blood and the MLNs. These data align with a decrease in immune activation, inflammation, and plasma VL in the same cohort as we previously reported (31).

The findings of this study should be considered in light of various limitations. We did not use phenotypic markers for CD8 T-cell differentiation, exhaustion, or markers of tissue-resident cells CD69 and CD103. Moreover, we did not provide functional cytotoxic assays to assess CD8 response. We are aware that the number of SIV-uninfected samples in MLNs was small compared to the rest of the groups, which might limit the power of statistical analysis, but we were unable to increase the number of samples because of the availability of the specimens. Finally, all MLN specimens in the untreated acute study group have been collected between days 29 to 60, which represents a steady-state acute phase, although frequencies of CD8 subsets in MLNs might be different in earlier infection, as well as the pick of viremia. Further studies to evaluate the dynamics of CD8 T cells in GALT are needed to confirm and expand upon our observations.

In summary, we present pieces of evidence indicating differential dynamics of effector and regulatory CD8 T cells during acute SIV infection and following very early ARV initiation in MLNs compared with blood. We demonstrated that despite the failure of very early ARV initiation in restoring the CD4/CD8 ratio in the blood and total CD8 Treg frequencies within the MLNs of treated animals, it could positively contribute to a better CD8 T-cell response by preserving the distribution and migratory potential within the CD8 compartment. These findings reinforce the benefits of early ARV initiation and the prominent role of CD8 T cells during SIV/HIV infections.

## MATERIALS AND METHODS

### Ethics statement.

All RMs were maintained at the nonhuman primate facilities of the Laval University, Quebec City, Canada, under the guidelines and recommendations of the Canadian Council on Animal Care (http://www.ccac.ca). Protocol and procedures were approved by the Laval University Animal Protection Committee (no. 106004). Animals with considerable signs of distress, disease, and weight loss were humanely euthanized, using an overdose of barbiturates, according to the guidelines of the Veterinary Medical Association.

### Experimental SIV infection protocol.

**FIG 6:**
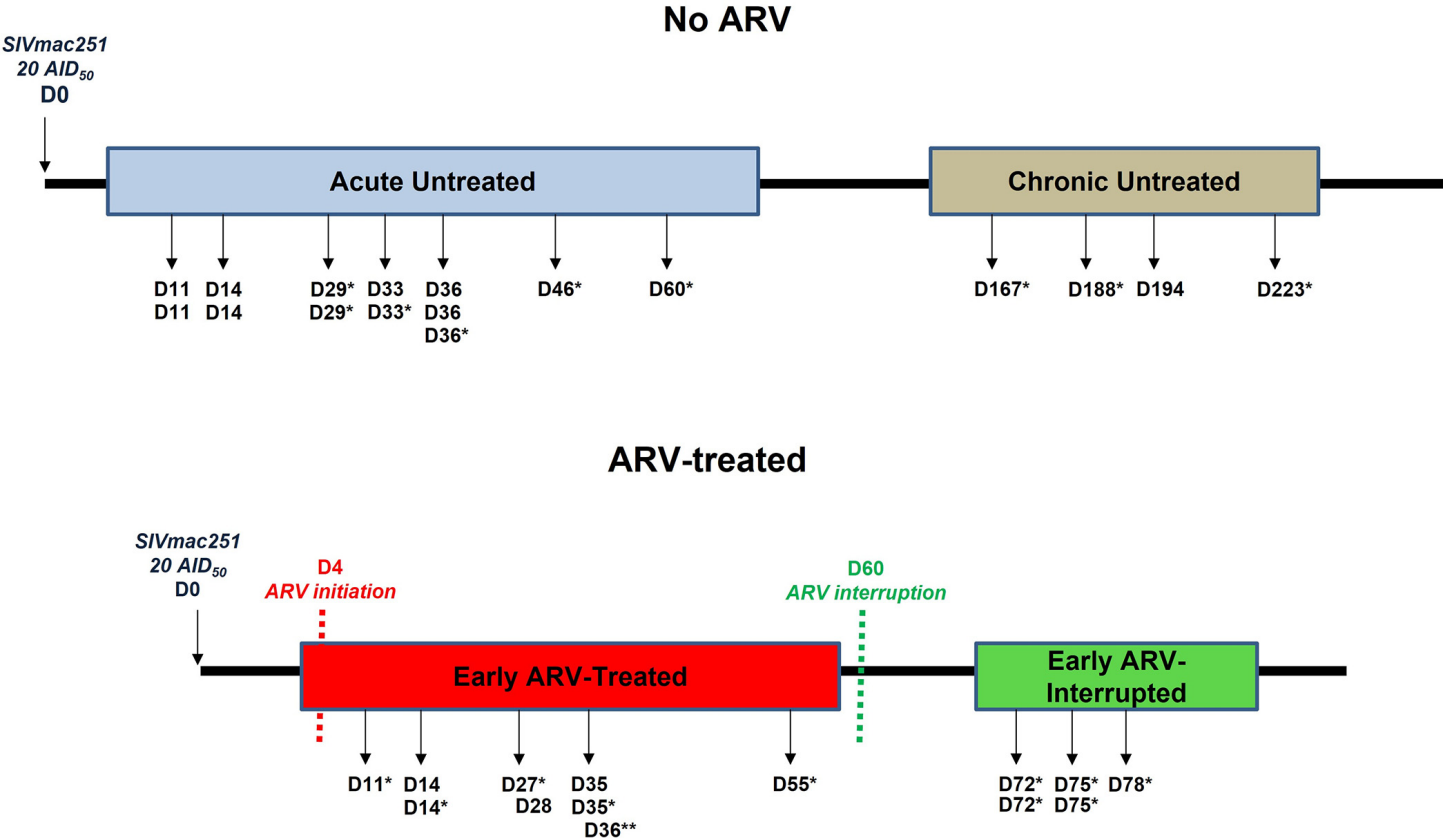
Study protocol. A total of 32 female RMs were enrolled in this study. A total of 25 animals were infected intravenously with 20 50% animal infectious doses of SIVmac251 virus, and the specimens were collected in both acute (early untreated) and chronic phases of infection. Nine monkeys were treated 4 days after the infection in a daily manner with an ARV cocktail. After 8 weeks of ARV treatment, the therapy was interrupted in five RMs. Two groups of nontreated animals in both acute (early untreated, *n* = 13) and chronic (*n* = 4) phases were also assessed. Blood specimens were obtained from 10 animals 3 days before the SIVmac251 infection to assess the uninfected baseline status. In addition, MLN specimens from two uninfected animals were also assessed to establish a baseline status in the MLNs. In the figure, black arrows represent the time point where samples from whole blood and/or mesenteric lymph nodes (MLNs) were taken.*, samples from MLNs were taken upon euthanasia; **, for one animal, only sample from MLNs was taken.

The infection protocol and study design are detailed in Fig. 6 and Table 1. In summary, a total of *n* = 32 female Chinese RMs were enrolled, and 25 animals were infected intravenously with 20 50% animal infectious dose (AID_50_) of SIVmac251 virus. Nine RMs were treated at day 4 postinfection with a cocktail of reverse transcriptase inhibitors tenofovir (20 mg/kg) and emtricitabine (20 mg/kg), protease inhibitors indinavir (2 mg/kg) and ritonavir (20 mg/kg), and integrase inhibitor raltegravir (20 mg/kg). To assess the impact of ARV interruption, in five RMs, ARV was interrupted after 8 weeks of treatment. Untreated SIV-infected RMs in acute (11 to 60 days postinfection; *n* = 13) and chronic (167 to 223 days postinfection; *n* = 4) phases, in addition to 10 uninfected RMs, were also included (Table 1). As described in Table 1, blood specimens from three untreated animals were assessed longitudinally in uninfected and early untreated groups, three other untreated animals were assessed longitudinally in both early untreated (acute) and chronic phases, and three early ARV-treated animals were assessed longitudinally in both early ARV-treated and early ARV-interrupted groups.

### Flow cytometric analysis.

Multiparameter flow cytometry was performed on whole blood and mechanically isolated MLN cells (31) using fluorochrome-conjugated antibodies as listed in Table 3. Dead cells were excluded from the analysis using the Live/Dead fixable aqua dead cell stain kit (Invitrogen, OR, USA). Following the extracellular staining, FoxP3 and Helios staining were performed using transcription factor buffer set (BD Bioscience). Flow cytometry acquisition was performed on a 3-laser BD Fortessa X-20 cytometer, and results were analyzed by FlowJo v10.2 (OR, USA).

**TABLE 3:** List of antibodies used for flow cytometry analysis

MAb^*a*^ and Fluorochrome	Clone	Company	Catalog no.
CD3-Alexa Fluor 488	SP34.2	BD Pharmingen	557705
CD3-Alexa Fluor 700	SP34.2	BD Pharmingen	557917
CD4-BV650	L200	BD Horizon	563737
CD8-APC-R-700	SK1	BD Horizon	565192
HLA-DR-BV605	G46-6	BD Pharmingen	562844
CD8beta-PE-cy7	SIDI88EE	eBioscience	25-5273-42
CD39-FITC	eBioA1	eBioscience	11-0399-42
CD39-APC	eBioA1	eBioscience	17-0399-42
CD45RA-APC-H7	5H9	BD Pharmingen	561212
CD161-BV421	DX12	BD Horizon	562615
CD183 (CXCR3)-PE-cy5	1C6/CXCR3	BD Pharmingen	551128
CD196 (CCR6)-PE	11A9	BD Pharmingen	551773
FoxP3-PE-CF594	236A/E7	BD Horizon	563955
TCR Vα7.2-APC	3C10	BioLegend	351708

a MAb, monoclonal antibody.

### Viral RNA quantification.

Viral loads in the sera of SIV-infected RMs were quantified by quantitative real-time PCR (RT-PCR) using a PureLink viral RNA/DNA kit (Invitrogen). Primers used were SIVmac-F, GCA GAG GAG GAA ATT ACC CAG TAC, and SIVmac-R, CAA TTT TA CCC AGG CAT TTA ATG TT. The probe used was SIVmac-Probe, 6FAM TGT CCA CCT GCC ATT AAG CCC GA TAMRA (6FAM, 6-carboxyfluorescein; TAMRA, 6-carboxytetramethylrhodamine). A plasmid encoding the *gag* gene of SIVmac251 was used as a standard. Amplifications were carried out with a 7500 real-time PCR system (Applied Biosystems) using the following parameters: 50°C for 5 min, 95°C for 20 s, and 40 cycles of 95°C for 15 s and 60°C for 1 min. Samples were run in duplicates, and results are expressed as SIV RNA copies/mL, with a limit of detection of 40 copies/mL (64).

### Statistical analysis.

GraphPad Prism v6.01 (CA, USA) was used for statistical analyses. Results are presented through the manuscript as medians with interquartile range. The Kruskal-Wallis test was performed to determine whether significant differences exist between the study groups. Differences among study groups were determined by the Mann-Whitney rank test of unpaired variables. Wilcoxon matched-pairs signed-rank test was used to compare paired variables. The Spearman test assessed correlations among study variables. Statistical significances (*P* ≤ 0.05) are shown in the figures.
